# The interaction between exercise and sleep with heart rate variability: cross-sectional study

**DOI:** 10.1007/s00421-025-05887-y

**Published:** 2025-07-05

**Authors:** Taylor Fein, T. Muhammad, Soomi Lee

**Affiliations:** 1 Integrative and Biomedical Physiology, Huck Institutes of Life Sciences, The Pennsylvania State University, 101 Huck Life Sciences Building, University Park, PA 16802 USA; 2 Department of Human Development and Family Studies, The Pennsylvania State University, 325 Health and Human Development Building, University Park, PA 16802 USA; 3 Center for Healthy Aging, The Pennsylvania State University, 219 Biobehavioral Health Building, University Park, PA 16802 USA

**Keywords:** Cardiovascular health, Autonomic function, Actigraphy, Physical activity, Insufficient sleep

## Abstract

**Purpose:**

To examine whether the interaction between exercise intensity and sleep duration/quality is associated with heart rate variability (HRV).

**Methods:**

A sample of 391 adults (*M*_age_ = 57 years) from the Midlife in the United States Biomarker study 2004–2009 provided sleep actigraphy, electrocardiogram (ECG) HRV measurements, completed the Pittsburgh Sleep Quality Index (PSQI), and answered questions on exercise habits. Participants were grouped as short (< 6 h) or non-short sleepers (≥ 6 h), poor (> 5 PSQI global score), or good sleepers (< 6 PSQI global score), and exercise was divided into vigorous (VPA) and moderate (MPA) intensities. Based on CDC guidelines, VPA was classified into adequate (≥ 75 min/week) and inadequate (< 75 min/week) groups. For MPA, adequate (≥ 150 min/week) and inadequate (< 150 min/week) groups. Linear models, adjusted for sociodemographic and health-related covariates, examined the interaction between sleep duration/quality and exercise on HRV.

**Results:**

Inadequate VPA was associated with lower HRV, HF-HRV (*B* = − 0.25, SE = 0.09, *p* = 0.007), and RMSSD (*B* = − 0.15, SE = 0.05, *p* = 0.0009). MPA showed no significant main associations with HRV. Sleep duration/quality did not show direct associations with HRV; however, interactions were found with sleep duration. Among short sleepers, inadequate VPA was associated with lower HF-HRV (*B* = − 0.62, SE = 0.25, *p* = 0.01) and inadequate MPA was associated with lower RMSSD (*B* = − 0.26, SE = 0.10, *p* = 0.01) compared to adequate exercise. Among non-short sleepers, there were no significant differences in HRV between exercise groups.

**Conclusion:**

These findings suggest that short sleep and inadequate exercise may interact to lower HRV.

**Supplementary Information:**

The online version contains supplementary material available at 10.1007/s00421-025-05887-y.

## Introduction

Heart rate variability (HRV) is a biomarker of autonomic function and the body’s ability to respond to stressors (Tiwari et al. 2021). Autonomic dysfunction, as measured by HRV, has been implicated in various pathologies, including metabolic syndrome, cardiovascular disease, diabetes, high blood pressure, and poor health outcomes (Wulsin et al. 2015). HRV has been linked to physical activity levels, such that physically active individuals exhibit higher HRV (Pichot et al. 2005; Zhong et al. 2005; Tseng et al. 2020). Another important lifestyle factor closely related to physical activity is sleep. Yet, the literature regarding the relationship between sleep and HRV is inconsistent. Some studies have reported that short sleep is associated with lower HRV (Sajjadieh et al. 2020; Vierra et al. 2022; Schlagintweit et al. 2023), while other studies have not found associations (Dettoni et al. 2012; Bourdillon et al. 2021). Moreover, the combined influence of exercise and sleep on HRV has not yet been explored.

The CDC recommends 150–300 min of moderate-intensity exercise per week, 75–150 min of vigorous-intensity aerobic activity per week, or a combination of both (Piercy et al. 2018). Yet, 46.3% of U.S. adults fail to reach the recommended guidelines for either aerobic and/or muscle-strengthening physical activity (Barnes and Schoenborn 2012), raising significant concerns about their cardiovascular health. In a large prospective study among older adults, researchers found that greater physical activity measures were associated with higher HRV measurements both cross-sectionally and 5 years later (Soares-Miranda et al. 2014). Additionally, in a sample of adults, total physical activity, leisure activity, and sport were associated with greater HRV (Tebar et al. 2020). In the Whitehall II study of civil servants, Rennie et al. 2003 found that increasing levels of vigorous physical activity (not moderate) were associated with higher HF-HRV in men, but not women. A systematic review on the impact of different exercise modalities on HRV in older adults found that endurance, coordination, and multimodal training, but not resistance training was associated with improvements in HRV (Grässler et al. 2021). Collectively, these findings show that greater physical activity improves HRV.

Insufficient or poor-quality sleep is associated with a wide range of health consequences, including an increased risk of developing diabetes, obesity, high blood pressure, cardiovascular disease, metabolic syndrome, poor mental health, depressed immune system, decreased cognitive performance, and increased all-cause mortality (Blackwell et al. 2006; Buxton and Marcelli 2010; Watson et al. 2015). The relationship between HRV and sleep remains unclear, as many studies have shown mixed results. For example, a cross-sectional study found no difference in HRV between participants who slept fewer than 6 h and those who slept 7 to < 9 h per night, while those sleeping 6 to < 7 h had significantly lower levels of parasympathetic nervous system (PNS) activity compared to 7 to < 9 h sleepers (Castro-Diehl et al. 2016). Similarly, poorer sleep quality was associated with lower HRV, though no significant relationship was found between HRV and self-reported sleep duration (Sajjadieh et al. 2020). Other studies, including one on older adults, found no significant differences in HRV between poor and good sleepers (Majeed et al. 2021; Nevels et al. 2023).

Previous literature has not examined the interactive associations of sleep and exercise with HRV but have explored sleep variables in response to exercise training. For example, Tseng et al. (2020) showed that three bouts of 40 min of aerobic exercise for 12 week intervention improved sleep quality and HRV compared to controls in middle-aged and older adults with poor sleep quality (82.5% female, *n* = 40). Participants were all poor sleepers (mostly postmenopausal female) who did not participate in regular physical exercise (defined by more than twice per week in the previous 3 months) before volunteering for study. They observed that the relationship between exercise intervention and increases in HRV may be mediated by increases in sleep quality (exercise improves HRV partially by improving sleep quality). Myllymäki et al. (2012) explored how varying exercise duration and intensity during the evening impacts HRV and sleep. Researchers did not find any differences in sleep duration/quality between exercise interventions, but nocturnal HRV was lower after 90-min exercise duration. Further exploration of this relationship is warranted with a larger sample of community-dwelling adults to examine whether the exercise–HRV relationship differs by sleep duration or sleep quality.

Sleep and exercise are important behaviors that influence HRV through physiological activity. Sleep is characterized by shifting autonomic dominance as evidence of fluctuation in HRV throughout sleep stages (Tobaldini et al. 2017). Non-rapid-eye-movement sleep (NREM) shows parasympathetic activity dominance and rapid eye movement sleep (REM) shows predominant sympathetic modulation indicating that sympatho-vagal balance shifts throughout the sleep cycle (Tobaldini et al. 2014). This suggests that sleep is important for autonomic regulation. During exercise, there is a withdrawal in parasympathetic and shift toward sympathetic activity to meet the metabolic demands of exercising muscle (Michael et al. 2017). This is represented by a decrease in HRV during exercise, while long-term exercise can improve autonomic tone (increasing vagal tone and decreasing sympathetic tone at rest) (Fu and Levine 2013). This is represented by higher HRV at rest for trained individuals compared to untrained individuals (Fu and Levine 2013).

Drawing on the restorative theory of sleep (Brinkman et al. 2025), we posit that short sleep duration or poor sleep quality may amplify the negative association of inadequate exercise with HRV. According to this theory, sleep is essential for cellular repair and normal daily biological function. Insufficient or poor-quality sleep disrupts these processes, increasing health risk (Cappuccio et al. 2010; Stenholm et al. 2019). In the context of the current study, short or poor sleep may amplify the negative association of inadequate exercise with HRV. Although studies exploring the interaction between sleep and exercise on health outcomes are limited, Liang et al. (2023) found curvilinear associations between sleep duration and mortality, with higher risks for older adults who had either short or long sleep durations. Importantly, these risks were mitigated among those who engaged in high volumes of physical activity or met recommended levels of moderate and vigorous physical activity (MVPA). Moreover, physical activity and sleep duration had additive and interactive associations with mortality risk. Specifically, older adults who did not meet MVPA guidelines and had short or long sleep durations were at greater risk of all-cause mortality compared to those who met MVPA guidelines and had normal sleep durations. These findings, along with the restorative theory of sleep (Brinkman et al. 2025), suggest that the combination of inadequate exercise and short sleep may be particularly detrimental to HRV.

In this study, we examined the relationship between exercise intensity and HRV and whether these associations vary based on sleep quality/duration. We hypothesize that the association between inadequate exercise and lower HRV would be more pronounced in individuals with short sleep duration/poor sleep quality. Investigating these associations may provide insights into how the health behaviors of exercise and sleep may interact and influence HRV.

## Methods

### Sample

Participants were selected from the Midlife in the United States (MIDUS) dataset, a national longitudinal study of health and well-being conducted at the University of Wisconsin-Madison (UW), Institute of Aging. A subset of 1255 adults were included in the Biomarker Study 2004–2009, where they traveled to General Clinical Research Centers (GCRC) (UW, UCLA, and Georgetown University) and stayed for a 2-day data collection protocol. Further information regarding the study visit can be found in Dienberg Love et al. (2010). On the second day, participants underwent baseline HRV measurements in the morning prior to a stressor test. Following the clinical visit, 441 participants from the UW site were invited to take part in a 7-day at-home actigraphy sleep assessment starting the Tuesday after their GCRC visit (Ryff et al. 2022). After excluding cases with missing data, 1050 participants were included in the analysis of HRV, exercise, and sleep quality, and 391 participants were included in the analysis of HRV, exercise, and sleep duration.

## Measures

### Exercise

The total weekly exercise was calculated from responses to the medical history questionnaire administered during the clinical visit. Participants were first provided with definitions of exercise intensity levels (Supplemental Table 1). This questionnaire captures various exercise domains, intensities, durations, and frequencies. For this analysis, we focused on moderate physical activity (MPA) and vigorous physical activity (VPA), consistent with the 2018 CDC physical activity guidelines (Piercy et al. 2018). Self-reported physical activity has been shown to be a reliable and valid method for association analyses in large-scale observational cohort studies (Warren et al. 2010). These measures are sufficient for categorizing individuals based on exercise engagement (Poirier 2014).

### Heart rate variability

HRV was assessed in the morning between 7 and 11 am of the second day of the clinical visit following a caffeine-free breakfast. Participants were in a seated position and instructed to remain quiet and breathe normally (Alen et al. 2021). Two baseline HRV measurements, each lasting 11 min, were recorded prior to the psychophysiology protocol. This protocol included a sequence of tasks—a cognitive stressor, a recovery period, and a second cognitive stressor, with each phase lasting 6 min. Beat-to-beat electrocardiogram (ECG) at a sampling rate of 500 Hz by a 16-bit National Instruments analog-to-digital (A/D) board installed in a microcomputer measured heart rate (HR) and several indices of HRV. Researchers visually inspected the data for artifacts and software errors to ensure the presence of normal R waves, and RR intervals. Only validated segments were used to calculate HRV indices and HR (Berntson et al. 1990). Root-mean-squared successive differences (rMSSD) and high-frequency HRV (HF-HRV; 0.15–0.50 Hz) were used as measures of parasympathetic activity in this analysis. These indices are established time and frequency markers, respectively (Shaffer and Ginsberg 2017). Higher values indicate greater PNS influence. RMSSD and HF-HRV were measured in two baseline measurements of 300-s (5-min) epochs and were natural log-transformed. Short-term (5-min) recordings of HRV have been established as a reliable and accurate method of measuring HRV (Malik 1996). Each HRV index was calculated as a mean of the two baseline measurements and were natural log-transformed. More information about the psychophysiology protocol can be found elsewhere (Ryff et al. 2022).

### Actigraphy-measured sleep duration

At the end of the GCRC visit, participants who opted into the 7-day sleep study were provided with a Mini Mitter Actiwatch®-64 device and detailed instructions for its use. The pre-programmed Actiwatch® activity monitor was worn on the non-dominant wrist and data were collected for 7 consecutive days. Participants mailed the device back through postage paid envelope and data were downloaded and stored in the Actiwatch® database for processing in the project office. A built-in motion sensor detects movements and can distinguish from wake/sleep times. The device was programmed to detect the number of movements in 30–2 epochs, and then, the total activity count was determined to be wake or sleep based on a predetermined wake value. If the total activity number was less than this threshold value, it was determined to be an epoch of sleep. We used total sleep time variable calculated as the total number of epochs between the Start Time and End time of the given interval scored as Sleep by Actiware software (Ryff et al. 2010). Participants who provided less than three nights of actigraphy sleep data were excluded from the analysis. Sleep duration was averaged across all available sleep nights. Short sleepers were classified as those who had < 6 h of total sleep time on average, while non-short sleepers were determined to be those who had ≥ 6 h of total sleep time on average. We did not include a separate category of long sleepers (> 9 h of total sleep time on average) because of the small cell size in our sample (*n* = 3).

### Self-reported sleep quality

Participants completed the Pittsburgh Sleep Quality Index (PSQI), a reliable and valid measure of sleep quality (Buysse et al. 1989). This index asks about participants’ perceptions about their habitual sleep across multiple dimensions, including sleep quality, sleep latency, sleep duration, habitual sleep efficiency, sleep disturbances, use of sleep medication, and daytime dysfunction. Each dimension is scored on a scale from 0 to 3 with higher score representing poorer sleep quality. The seven components are weighed equally and summed to create a global sleep score ranging from 0 to 21 with higher scores representing worse overall sleep quality. The global score of > 5 has been used clinically to determine poor sleep quality (Buysse et al. 1989). Thus, participants with a score > 5 were classified as poor sleepers, while those with a score equal to or below 5 were classified as good sleepers.

### Covariates

Socio-demographic and health covariates that are known to be related to HRV and sleep were controlled for in our analysis. These included age, sex, marriage status, race, education, chronic conditions, body mass index (BMI), depressive symptoms, and smoking status. Age was determined by subtracting the date of the clinic visit from the participants’ date of birth. Sex was coded as 0 = men and 1 = women, marriage status was coded as 0 = married and 1 = not married, and race was coded as 0 = non-Hispanic white and 1 = non-white. Education was on a scale from 1 to 12 ranging from 1 = No school or some grade school, 2 = Junior high school, 3 = Some high school … 11 = Master’s degree, and 12 = Doctoral or other professional degree. Total number of chronic conditions (ever) was a constructed variable tallied based on the total number of “Yes” responses in the medical history questionnaire about ever diagnoses of illnesses/conditions. BMI was calculated from the participant’s height and weight recorded at the clinic visit. Depressive symptoms were assessed using the Center for Epidemiological Studies Depression (CESD). The CESD score was treated as a continuous variable ranging from 0 to 60, with higher scores indicating greater levels of depressive symptoms. Smoking was coded as 0 = never smoker, 1 = past smoker, and 2 = current smoker.

### Statistical analysis

Sample descriptive statistics and tests of differences were used to compare exercise groups using the Wilcoxon test for continuous variables and chi-squared test for categorical variables. Three sets of analyses were conducted to contextualize the exercise and HRV relationship. First, to determine potential dose–response relationships, VPA and MPA were used as continuous exercise minute variables. Second, exercise was further categorized into levels in a graded spline model. For VPA, the groups were defined as: inactive (0 min), insufficiently active (1–74 min), active (75–149 min), and highly active (≥ 150 min). For MPA, the categories were: inactive (0 min), insufficiently active (1–149 min), active (150–299 min), and highly active (≥ 300 min). Third, to assess this relationship with public health guidelines, binary groups of MPA and VPA were analyzed. MPA < 150 min weekly was categorized as inadequate, while 150 min or more of MPA weekly was categorized as adequate. Similarly, VPA < 75 min was categorized as inadequate and ≥ 75 min as adequate (Piercy et al. 2018). Linear models were used to examine differences in the levels of each HRV measure by exercise or sleep and their interactions. Model 1 included only exercise variables (continuous, graded, or binary) or sleep (either duration or quality) to examine unadjusted associations. Model 2 added sociodemographic covariates (i.e., age, sex, marital status, race, and education). Model 3 added health-related covariates (i.e., chronic conditions, BMI, CESD, and smoking status) to examine fully adjusted associations. A further analysis examined the interactive associations of exercise and sleep duration/quality with HRV, adjusting for all covariates. To assist with interpretation and practical application, we used binary exercise group variables in all interaction analyses. Additionally, two sets of supplemental analysis were conducted to explore non-linear relationships and potential sex differences. Prior literature has highlighted sex differences in HRV as evidenced by greater vagal activity in females (Koenig and Thayer 2016). All our data had < 10% missingness which suggests it is less prone to bias and unlikely to impact our statistical power (Bennett 2001). Data were analyzed with *emmeans* and *ggplot2*/*gtsummary* packages for tables/figures in R statistical software (Rversion 4.4.1, http://www.r-project.org). *Emmeans* package was utilized for running pairwise comparisons of means for all combinations of sleep and exercise variables. Statistical significance was determined at *p* < 0.05.

## Results

### Descriptive statistics

Descriptive statistics are shown in Table 1, comparing exercise groups with pairwise comparisons. Age ranged from 35 to 86 (*M* = 57; SD = 12), 43% male, 77% non-Hispanic white, and education *M* = 3+ years of college with no degree. There were significant differences between adequate VPA and inadequate VPA groups in age, sex, sex, marital status, race, education, number of chronic conditions, BMI, CESD scores, RMSSD, and HF-HRV. The two groups did not differ by smoking status, sleep duration, and sleep quality. There were also significant differences between adequate MPA and inadequate MPA groups in age, sex, sex, marital status, race, education, number of chronic conditions, BMI, CESD scores, smoking status, and sleep quality. There were no significant differences between adequate MPA and inadequate MPA groups in smoking status, sleep duration, RMSSD, and HF-HRV. A comparison between those who provided HRV data and those who did not are included in (Supplemental Table 2). Those who provided HRV data were younger, had fewer chronic conditions, and were more likely to engage in VPA, compared to those who did not.

**Table 1:** Sample descriptives and tests of group differences between VPA and MPA (*N* = 1,255)

		Adequate	Inadequate		Adequate	Inadequate	
	Overall	VPA	VPA		MPA	MPA	
Characteristic	*N* = 1255^1^	*N* = 256^1^	*N* = 999^1^	*p *value^2^	*N* = 495^1^	*N* = 757^1^	*p *value^2^
Age (years)	57 (12)	54 (10)	58 (12)	<** 0**.**001**	58 (12)	57 (12)	**0**.**033**
Min, max	35, 86	36, 83	35, 86		35, 86	37, 86	
Sex				**0**.**006**			<** 0**.**001**
Male	542 (43%)	130 (51%)	412 (41%)		245 (49%)	297 (39%)	
Female	713 (57%)	126 (49%)	587 (59%)		250 (51%)	460 (61%)	
Marriage				**0**.**009**			**0**.**001**
Married	789 (63%)	179 (70%)	610 (61%)		338 (68%)	448 (59%)	
Unmarried	466 (37%)	77 (30%)	389 (39%)		157 (32%)	309 (41%)	
Race				<** 0**.**001**			<** 0**.**001**
Non-Hispanic White	959 (77%)	219 (86%)	740 (74%)		408 (82%)	549 (73%)	
Non-white	291 (23%)	36 (14%)	255 (26%)		87 (18%)	204 (27%)	
Level of education	7.47 (2.53)	8.08 (2.42)	7.32 (2.54)	<** 0**.**001**	7.67 (2.56)	7.34 (2.51)	**0**.**021**
Chronic conditions	3.37 (2.48)	2.60 (2.01)	3.56 (2.55)	<** 0**.**001**	3.11 (2.29)	3.54 (2.59)	**0**.**012**
BMI (kg/m^2^)	30 (7)	28 (6)	30 (7)	<** 0**.**001**	29 (6)	30 (7)	**0**.**001**
CESD	9 (8)	7 (7)	9 (8)	<** 0**.**001**	8 (8)	9 (9)	**0**.**003**
Smoker				0.2			0.12
No	658 (52%)	143 (56%)	515 (52%)		261 (53%)	394 (52%)	
Former	409 (33%)	82 (32%)	327 (33%)		172 (35%)	237 (31%)	
Yes	187 (15%)	30 (12%)	157 (16%)		62 (13%)	125 (17%)	
Sleep duration				0.3			0.9
Non-short	262 (59%)	42 (54%)	220 (61%)		88 (60%)	174 (59%)	
Short	179 (41%)	36 (46%)	143 (39%)		59 (40%)	120 (41%)	
Sleep quality				0.2			**0**.**003**
Good	608 (52%)	136 (56%)	472 (51%)		266 (57%)	340 (48%)	
Poor	564 (48%)	107 (44%)	457 (49%)		200 (43%)	363 (52%)	
RMSSD ln(ms)	2.92 (0.63)	3.05 (0.65)	2.88 (0.62)	<** 0**.**001**	2.90 (0.59)	2.93 (0.66)	0.8
HF-HRV ln(ms^2^)	4.79 (1.28)	5.04 (1.31)	4.72 (1.26)	<** 0**.**001**	4.76 (1.23)	4.81 (1.31)	0.9

All HRV measures were natural log-transformed

Bold *p*-values indicate statistical significance (*p* < 0.05)

*CESD* Center for Epidemiological Studies Depression, *BMI* body mass index, *kg* kilograms, *m* meters, *MVPA* moderate-to-vigorous intensity physical activity, *VPA* vigorous physical activity, *MPA* moderate physical activity, *HF-HRV* high-frequency HRV; *RMSSD* root-mean-squared successive differences, *ln* natural log, *ms* milliseconds

^1^mean (SD) for continuous; *n* (%) for categorical

^2^Wilcoxon rank sum test; Pearson’s Chi-squared test

### Main associations of exercise with HRV

**Fig. 1:**
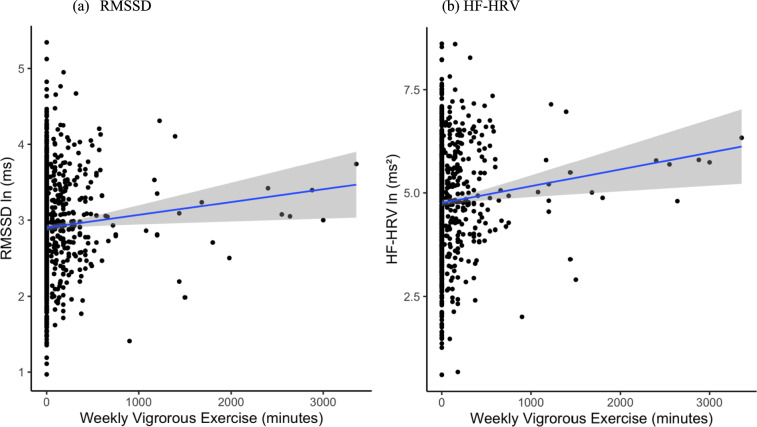
Individual data points of **a** RMSSD and **b** HF-HRV by VPA minutes (*N* = 1138)

Figure 1 shows the significant association of VPA exercise minutes with HRV indices (*B* = 0.0003, SE = 0. 0001, CI [0.00005, 0.0006], *p* = 0.021 for HF-HRV; *B* = 0.0001, SE = 0.00007, CI [0.000002, 0.0002], *p* = 0.046 for RMSSD). There were no significant associations between MPA minutes with HRV. For VPA graded spline model, significant main effects were found only in the highly active group for both HRV indices (RMSSD: *B* = 0.179, SE = 0.51, CI [0.078, 0.28], *p* = 0.001, HF-HRV: *B* = 0.343, SE = 0.103, CI [0.14, 0.55], *p* = 0.001). MPA showed no significant effects. In the binary exercise models, inadequate VPA was associated with a 0.25 ln (ms^2^) decrease in HF-HRV (*B* = − 0.25, SE = 0.09, 95% CI [− 0.43, − 0.07], *p* = 0.007), which is comparable in magnitude to an 8-year difference in age (*B* = − 0.03, SE = 0.004, 95% CI [− 0.04, − 0.02], *p* < 0.001). Inadequate VPA was associated with a 0.15 ln (ms) decrease in RMSSD (*B* = − 0.15, SE = 0.05, 95% CI [− 0.25, − 0.06], *p* < 0.001), which is comparable in magnitude to a 15-year difference in age (*B* = − 0.01, SE = 0.001, 95% CI [− 0.01, − 0.003], *p* < 0.001). There were no significant main associations of MPA groups with HRV. Tables 2 and 3 present the results from the linear models examining the associations of VPA and MPA groups on RMSSD and HF-HRV.

**Table 2:** Linear model results on the association between VPA and HRV (*N* = 1138)

	RMSSD ln (ms)			HF-HRV ln (ms^2^)		
Characteristic	Beta	95% CI^1^	*p *value	Beta	95% CI^1^	*p *value
Model 1: unadjusted						
Inadequate VPA	− 0.16	− 0.25, − 0.07	<** 0**.**001**	− 0.3	− 0.51, − 0.14	**0**.**001**
Model 2: partially adjusted						
Inadequate VPA	− 0.16	− 0.25, − 0.07	**0**.**001**	− 0.3	0.45, 0.09	**0**.**003**
Age (years)	− 0.01	− 0.01, − 0.01	<** 0**.**001**	− 0.03	− 0.04, − 0.03	<** 0**.**001**
Non-white	0.20	0.11, 0.30	<** 0**.**001**	0.40	0.21, 0.58	<** 0**.**001**
Female	0.01	− 0.07, 0.08	0.9	0.12	− 0.03, 0.27	0.1
Unmarried	0.04	− 0.03, 0.12	0.3	0.04	− 0.12, 0.20	0.6
Education	− 0.01	− 0.02, 0.01	0.5	− 0.01	− 0.04, 0.02	0.4
Model 3: adjusted						
Inadequate VPA	− 0.15	− 0.24, − 0.06	**0**.**001**	− 0.25	− 0.43, − 0.07	**0**.**007**
Female	0.02	− 0.05, 0.10	0.6	0.15	0.00, 0.30	**0**.**045**
Age (years)	− 0.01	− 0.01, − 0.00	<** 0**.**001**	− 0.03	− 0.04, − 0.02	<** 0**.**001**
Non-white	0.20	0.11, 0.29	<** 0**.**001**	0.41	0.23, 0.50	<** 0**.**001**
Unmarried	0.03	− 0.05, 0.11	0.5	0.03	− 0.13, 0.19	0.7
Education	0.00	− 0.02, 0.01	0.9	− 0.01	− 0.04, 0.02	0.6
Chronic conditions	− 0.01	− 0.03, 0.01	0.2	− 0.03	− 0.06, 0.01	0.14
CESD	− 0.00	− 0.01, 0.00	0.8	− 0.01	− 0.02, 0.00	0.077
Former smoker	− 0.01	− 0.09, 0.08	0.9	0.00	− 0.16, 0.17	> 0.9
Current smoker	0.15	0.04, 0.26	**0**.**01**	0.31	0.08, 0.54	**0**.**007**
BMI	− 0.00	− 0.01, 0.01	0.8	− 0.01	− 0.02, 0.01	0.4

All HRV measures were natural log-transformed

Bold *p*-values indicate statistical significance (*p* < 0.05)

*VPA* vigorous physical activity, *CESD* Center for Epidemiological Studies Depression, *BMI* body mass index, *B* unstandardized beta coefficient, *CI* confidence interval, *HF-HRV* high-frequency HRV, *RMSSD* root-mean-squared successive differences, *ln* natural log, *ms* milliseconds

**Table 3:** Linear model results on the association between MPA and HRV (*N* = 1136)

	RMSSD ln (ms)			HF-HRV ln (ms^2^)		
Characteristic	Beta	95% CI^1^	*p *value	Beta	95% CI^1^	*p *value
Model 1: unadjusted						
Inadequate MPA	0.03	− 0.04, 0.11	0.4	0.05	− 0.11, 0.20	0.6
Model 2: partially adjusted						
Inadequate MPA	0.00	− 0.07, 0.07	> 0.9	− 0.05	− 0.20, 0.10	0.5
Age (years)	− 0.01	− 0.01, − 0.01	<** 0**.**001**	− 0.03	− 0.04, − 0.03	<** 0**.**001**
Non-white	0.19	0.10, 0.28	<** 0**.**001**	0.37	0.19, 0.56	<** 0**.**001**
Female	0.00	− 0.08, 0.07	> 0.9	0.11	− 0.04, 0.26	0.2
Unmarried	0.04	− 0.04, 0.12	0.3	0.04	− 0.12, 0.20	0.6
Education	0.00	− 0.02, 0.01	0.8	− 0.01	− 0.04, 0.02	0.6
Model 3: adjusted						
Inadequate MPA	0.00	− 0.07, 0.08	> 0.9	− 0.03	− 0.18, 0.11	0.6
Female	0.01	− 0.06, 0.09	0.7	0.14	− 0.01, 0.29	0.069
Age (years)	− 0.01	− 0.01, 0.00	<** 0**.**001**	− 0.03	− 0.04, − 0.02	<** 0**.**001**
Non-white	0.19	0.10, 0.28	<** 0**.**001**	0.40	0.21, 0.58	<** 0**.**001**
Unmarried	0.03	− 0.05, 0.11	0.5	0.04	− 0.12, 0.20	0.7
Education	0.00	− 0.01, 0.02	0.8	0.00	− 0.04, 0.03	0.7
Chronic conditions	− 0.01	− 0.03, 0.01	0.2	− 0.03	− 0.06, 0.01	0.13
CESD	0.00	− 0.01, 0.00	0.6	− 0.01	− 0.02, 0.00	0.054
Former smoker	0.00	− 0.08, 0.08	> 0.9	0.01	− 0.15, 0.18	0.9
Current smoker	0.14	0.03, 0.26	**0**.**013**	0.30	0.08, 0.53	**0**.**009**
BMI	0.00	− 0.01, 0.00	0.6	− 0.01	− 0.02, 0.00	0.3

All HRV measures were natural log-transformed

Bold *p*-values indicate statistical significance (*p* < 0.05)

*MPA* moderate physical activity, *CESD* Center for Epidemiological Studies Depression, *BMI* body mass index, *B* unstandardized beta coefficient, *CI* confidence interval, *HF-HRV* high-frequency HRV, *RMSSD* root-mean-squared successive differences, *ln* natural log, *ms* milliseconds

### Main associations of sleep duration and sleep quality with HRV

**Fig. 2:**
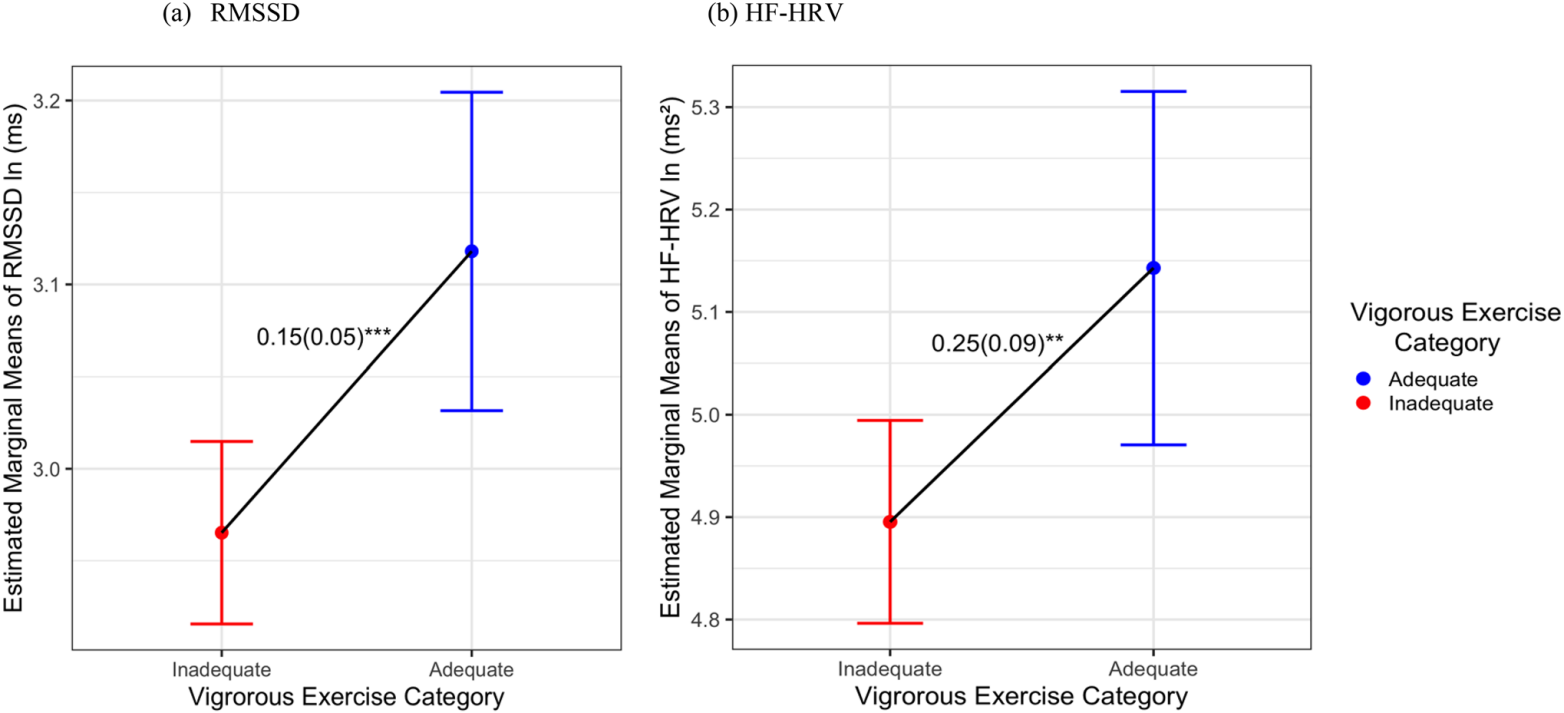
Comparing estimated marginal means of **a** RMSSD and **b** HF-HRV by VPA (*N* = 1138). *HF-HRV* high-frequency HRV, *RMSSD* root-mean-squared successive differences, *ln* natural log, *ms* milliseconds; all HRV measures were natural log-transformed; **p < 0.01, ***p < 0.001

**Fig. 3:**
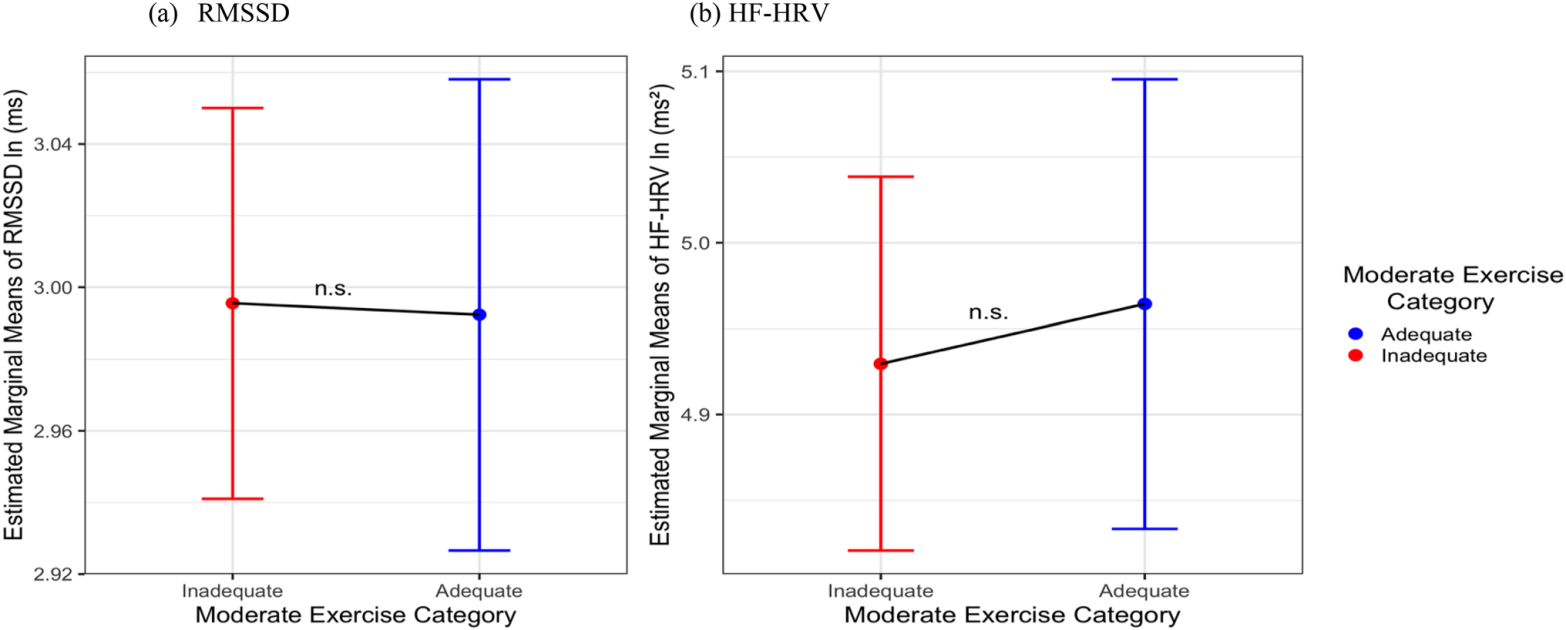
Comparing estimated marginal means of **a** RMSSD and **b** HF-HRV by MPA (*N* = 1138). *HF-HRV* high-frequency HRV, *RMSSD* root-mean-squared successive differences, *ln* natural log, *ms* milliseconds; all HRV measures were natural log-transformed

There were no significant main associations of sleep duration or sleep quality with HRV across all models. Figures 2 and 3 show the main effects of VPA and MPA on HRV. Supplemental Tables 3 and 4 present the linear models results including sleep duration and sleep quality and HRV.

### Interactive associations of exercise intensity and sleep duration/sleep quality with HRV

**Fig. 4:**
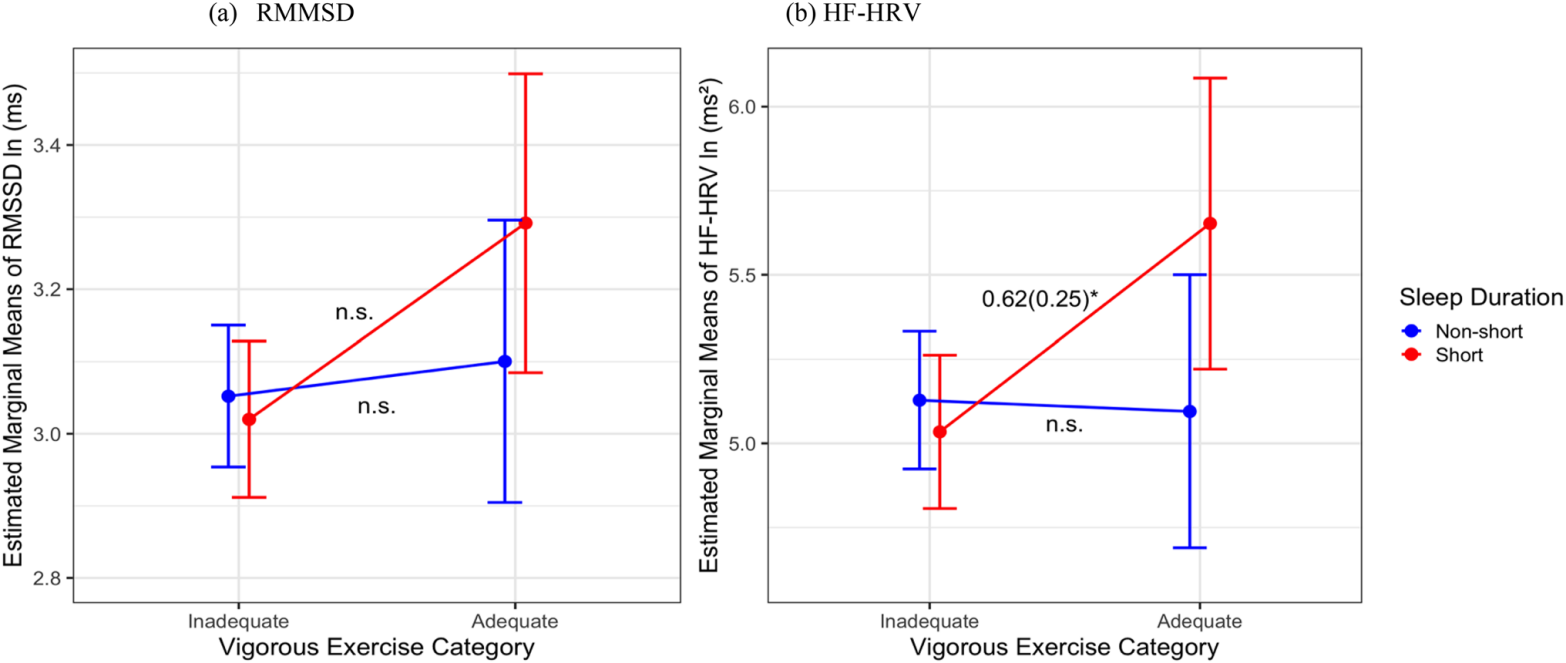
Interaction between VPA and sleep duration with **a** RMSSD and **b** HF-HRV (*N* = 391). *HF-HRV* high-frequency HRV, *RMSSD* root-mean-squared successive differences, *ln* natural log, *ms* milliseconds; all HRV measures were natural log-transformed; *n*.*s*. not significant. *p < 0.05

**Fig. 5:**
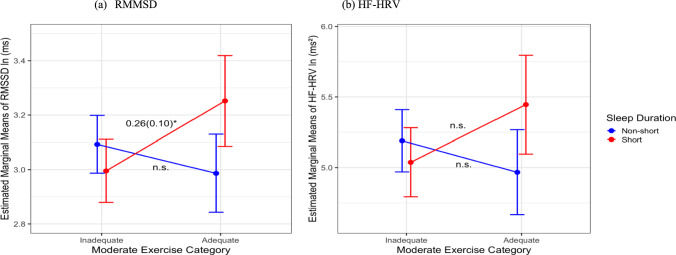
Interaction between MPA and sleep duration with **a** RMSSD and **b** HF-HRV (*N* = 391). *HF-HRV* high-frequency HRV, *RMSSD* root-mean-squared successive differences, *ln* natural log, *ms* milliseconds; all HRV measures were natural log-transformed; *n*.*s*. = not significant. *p < 0.05

There were significant interactions found between VPA/MPA and sleep duration with the two HRV metrics illustrated in Figs. 4 and 5, but no interactions found between MPA/VPA groups and sleep quality with HRV. Figure 3 shows that for HF-HRV, among short sleepers, adequate VPA was associated with higher HF-HRV indices (*B* = 0.62, SE = 0.25, CI [− 1.29, − 0.02], *p* = 0.01) compared to engaging in inadequate VPA, while there were no significant interactions for RMSSD (*B* = 0.22, SE = 0.16, CI [− 0.53, 0.08], *p* = 0.15). Figure 5 shows that for RMSSD, among short sleepers, adequate MPA was associated with higher RMSSD (*B* = 0.26, SE = 0.10, CI [− 0.62, 0.11], *p* = 0.01), while there were no significance for HF-HRV (*B* = 0.41, SE = 0.22, CI [− 1.16, − 0.10], *p* = 0.058) compared to engaging in inadequate MPA. Among the non-short sleeper group, there were no significant differences in HRV between exercise groups.

### Supplemental analyses

A potential non-linear relationship between exercise and HRV was tested by adding a quadratic term for the continuous predictors of moderate, vigorous, and sleep minutes. A significant quadratic effect was observed for vigorous exercise minutes on RMSSD (*B* = − 1.30e−7, *SE* = 6.5e−8, 95% CI [− 2.59e−7, − 2e−9], *p* = 0.046), indicating a modest curvilinear relationship. All other models with quadratic terms were nonsignificant.

To determine whether results are impacted by sex differences, an interaction model between MPA/VPA groups and sex were tested for both HRV indices. All models with the interaction term between MPA or VPA and sex were nonsignificant.

### Sensitivity analysis

A recall–bias sensitivity test was conducted on self-reported physical activity data with ± 20% error was ran to assess the robustness of findings. VPA showed the same trends, when VPA was reduced by 20%, and the association was statistically significant but slightly reduced (HF-HRV: *B* = − 0.23, SE = 0.10, 95% CI [− 0.42, − 0.04], *p* = 0.018, RMSSD: *B* = − 0.14, SE = 0.05, 95% CI [− 0.23, − 0.05], *p* = 0.003). When VPA was increased by 20%, the association was statistically significant and nearly identical to the main analysis (HF-HRV: *B* = − 0.25, SE = 0.09, 95% CI [− 0.43, − 0.07], *p* = 0.006, RMSSD: *B* = − 0.15, SE = 0.05, 95% CI [− 0.24, − 0.06], *p* = 0.001). There remained no significant associations between MPA and HRV.

## Discussion

This study examined the interactive associations of exercise and sleep with HRV. There were main associations between VPA and HRV, with the adequate VPA group showing higher HRV than the inadequate VPA group. In supplemental analysis, when stratified by inactive, insufficiently active, active, and highly active VPA, only the highly active group was associated with HRV. There were no main associations between MPA and HRV. Sleep alone was not significantly associated with HRV in this sample; short and non-short sleepers and poor and good quality sleepers did not differ in HRV. However, there were interactive associations between sleep duration and exercise with HRV. Among short sleepers, those who were in the adequate VPA group had a significantly higher HF-HRV than those who were in the inadequate VPA group. Among short sleepers, those who were in the adequate MPA group had a significantly higher RMSSD than those who were in the inadequate MPA group. There were no differences in HRV by VPA or MPA group in non-short sleepers.

A lower HRV has been associated with increased risk of cardiovascular disease, while a higher HRV is a marker of cardiovascular health (Tiwari et al. 2021). Exercise is a known behavior to promote cardiovascular health and autonomic function. Many studies have shown that exercise training can increase HRV due to greater parasympathetic activity at rest (Soares-Miranda et al. 2014; Tebar et al. 2020). Our findings suggest that engaging in the recommended amount of vigorous exercise may offer benefits to HRV. VPA is known to produce greater benefits to cardiovascular fitness than moderate exercise. The autonomic neural hypothesis posits that endurance training causes greater vagal tone at rest leading to decreases in resting heart rate. Aerobic training also alters autonomic function by decreasing sympathetic activity at rest (Swain and Franklin 2006). This hypothesis is supported by increases in HRV observed following exercise training programs (specifically of vigorous intensity) (Levy et al. 1998; Billman et al. 2015). Ferreira et al. (2017) found that intensity is an important aspect of an exercising training program to induce changes in HRV. A cross-sectional study showed that HF-HRV was higher in endurance trained compared to sedentary individuals, but there were no significant differences between the sedentary and moderate exercise groups among older adults (Monahan et al. 2000). Cardiovascular adaptations associated with VPA may be necessary to show significant improvements in HRV. In the current study, exercise was stratified into four levels of activity, and we observed significant main effects in the highly active group for vigorous exercise on both HRV indices, while moderate exercise levels did not show significant effects. This suggests that the highly active group may be driving the significant association between VPA and HRV. Lee et al. (2022) found that individuals who participated in 150–300 min of vigorous physical activity per week showed maximal reductions in mortality risk. The potential benefits to HRV—particularly when exceeding the weekly recommendation—may help explain this reduction in mortality.

The absence of main associations between HRV and sleep duration or quality aligns with findings from several previous studies. For example, Sajjadieh et al. (2020) reported no significant associations between sleep duration and HRV parameters, while Nevels et al. (2023), and Rocha et al. (2023) found no correlations between HRV and PSQI subscales in participants at rest. However, HRV may have more proximal associations with sleep on a daily basis. On days nights when participants sleep shorter or poorer, they may exhibit lower HRV. Supporting this, a few randomized-controlled trials have shown that HRV decreases during nights of sleep restriction compared to baseline sleep nights. For instance, Schlagintweit et al. (2023) observed significantly lower nighttime HRV during nights with partial sleep restriction compared to baseline nights in middle-aged men. Similarly, Bourdillon et al. (2021) found that HRV was reduced during 3-h sleep nights compared to baseline in healthy subjects. Together, these findings suggest that HRV may have more immediate, nightly associations with sleep, which can be difficult to capture in cross-sectional studies.

The interaction between sleep duration and exercise intensity is a novel finding. Among short sleepers, those who engaged in adequate exercise had significant higher HRV than those did not. The sensitivity of these associations varied by exercise intensity and HRV index. For MPA, a significant interaction was observed for RMSSD, whereas for VPA, the interaction was significant for HF-HRV. High-intensity interval training has been shown to be the most effective at improving RMSSD (Yang et al. 2024) which may explain why vigorous exercise is associated with greater RMSSD regardless of sleep duration. In the current study, higher RMSSD was observed in the adequate MPA group among short sleepers. While RMSSD reflects vagal changes in HRV and is correlated with HF-HRV, the former may be more susceptible to contextual influences (Shaffer et al. 2014). Future research should explore the differential effects of exercise intensity on HRV across diverse populations, taking into account potential contextual modifiers. There were no differences in HRV by exercise in the non-short sleeper group. Sleeping ≥ 6 h may play a protective role in the association of inadequate exercise with poorer HRV. On the other hand, short sleep may impair the restorative processes involved in sleep (Brinkman et al. 2025) and make individuals more susceptible to the negative associations of inadequate exercise and HRV. Sleep deprivation may contribute to imbalances in the autonomic nervous system (ANS), as illustrated by a lower HRV associated with inadequate exercise. Yet, the restorative process of sufficient sleep may maintain homeostatic dynamic balance of the ANS and moderate the negative relationship between inadequate exercise and HRV. These findings align with Liang et al. (2023) who also found that short sleepers and not recommended MVPA had the highest all-cause and cardiovascular mortality risks. This suggests a synergistic association of short sleep and inadequate exercise with negative health outcomes. Yet, sleep quality and exercise did not show any significant interactions with HRV. This may be due to a relatively healthy study sample with majority reported good sleep quality (52%). The relationship between sleep quality and HRV may be dependent on the individual’s overall health (Rocha et al. 2023). The measure used for sleep quality may also not capture the day-to-day differences in sleep quality.

Sufficient, high-quality sleep is essential for the maintenance of homeostatic function and autonomic cardiovascular regulation, but may be vulnerable to disorders of autonomic dysfunction (Tobaldini et al. 2013; Miglis 2017). Studies that have induced acute sleep deprivation in individuals have shown increases in sympathetic activity with decreases in both parasympathetic activity and baroreflex sensitivity leading to changes in cardiovascular reactivity (Yang et al. 2021). HRV is an indicator of the body’s ability to respond to stressors and adapt to the internal/external demands it may encounter (Shaffer et al. 2014). Exercise improves ANS adaptation by increasing cardiac vagal tone, while decreasing sympathetic activity at rest (Fu and Levine 2013). Individually, when exhibiting either sufficient sleep or adequate exercise, the body may be able to counteract the impacts on autonomic function from either short sleep or inadequate exercise. However, the combination of short sleep and inadequate exercise may leave the body vulnerable and unable to physiologically adapt to both stressors. Thus, when sleep is insufficient and exercise is inadequate, there may be an additive effect to produce pathophysiology on the human body. These two poor health behaviors may impair the body’s ability to adapt and contribute to autonomic imbalance as evidenced by lower HRV.

## Implications

About half of older adults report sleep problems, which contribute to autonomic dysfunction (Tatineny et al. 2020). This autonomic impairment also aggravates sleep disturbances creating a negative feedback loop (Kim et al. 2022). The natural variations of sympathetic activity during REM sleep and parasympathetic activity during NREM sleep are important for autonomic regulation. Autonomic dysfunction can lead to alterations in baroreflex activity and elevated norepinephrine (NE) that may contribute to sleep disturbances. Sleep deprivation is associated with hyperarousal via the hypothalamus which contributes to sympathetic nervous system (SNS) overactivity and autonomic complications.

This population-level analysis highlights the need for future mechanistic research. Short-term HRV is generated by the dynamic relationship of the SNS and PNS as well as heart rate regulatory mechanisms including respiratory sinus arrhythmia, baroreceptor activity, and vascular tone (Shaffer and Ginsberg 2017). Examining the two negative health behaviors of short sleep and inadequate exercise reveals the additive negative association of these behaviors with HRV. The combination of physical inactivity and short sleep may disrupt autonomic function and thus contribute to this association. Several neuromodulators, such as vagus nerve stimulation and carotid baroreceptor stimulation, have been posed to help correct sympathetic overactivity, increase baroreceptor sensitivity, and improve sleep disorders (Kim et al. 2022). The mechanisms in which these neuromodulators improve sleep and autonomic function may also be stimulated by exercise training. Aging causes declines in baroreflex control which decreases cardiac autonomic function (Kim et al. 2022). Exercise has been shown to prevent and improve these changes by increasing baroreflex sensitivity (BRS). Augmentation in arterial elasticity, muscarinic receptor density, and integration of sensory signals in the nucleus tractus solitarius (NTS) are mechanisms that contribute to improvements in BRS (Madden et al. 2010; La Rovere and Pinna 2014). Improvements in BRS through exercise training may counteract sympathetic overactivity by decreasing circulating NE levels (Mueller 2007). On the other hand, physical inactivity contributes to SNS excitation potentially through alterations in rostral ventrolateral medulla (RVLM) neurons (Mueller 2007; Mischel and Mueller 2011). Improvements in autonomic function through exercise may be the mechanism which explains this interaction. Thus, exercise training may be a beneficial intervention to counteract autonomic dysfunction associated with short sleep duration and improve HRV. Experimental research is needed to parse out the specific physiological underpinnings of these observed relationships.

## Strength and limitations

There are several strengths to this study. Subjective and objective measurements of sleep were included to help capture different aspects of one’s sleep quantity and quality. The HRV measurements were based on a validated and reliable tool. The use of a national representative sample of U.S. adults increases the generalizability of the results. There are, however, some limitations to this work. Exercise was self-reported and may be prone to recall bias. In MIDUS, actigraphy-measured sleep data are available, but the specific actigraphy device used in the MIDUS study (Mini Mitter Actiwatch®-64 activity monitor) was designed to detect wake versus sleep. MIDUS uses a 30 s sample epoch and a set wake threshold value of 40 based on total activity counts to differentiate wake versus sleep. Thus, it was not intended to detect physical activity context/intensity (light versus moderate versus vigorous activity) and validated devices for exercise detection should be utilized in the future*.* It may also be beneficial to observe how exercise modality (e.g., resistance training vs. aerobic exercise) may impact the exercise, sleep, and HRV relationship. Short-term and lab-based HRV measurements might have been influenced by other confounds (e.g., whether participant is stressed during measurements and/or prior day activity levels) and may cause reduced HRV that does not reflect HRV in everyday conditions (Chen et al. 2011; Kim et al. 2018; Peabody et al. 2023). Additionally, pre-protocol fluid intake was not standardized, and greater water consumption may cause increases in HRV (Routledge et al. 2002). HRV is also subject to individual variability and influenced by a person’s genetics, lifestyle factors, stress levels, seasonal variation, and training status (Kim et al. 2018; Damoun et al. 2024). Thus, future studies should explore within person variability by incorporating repeated measures of HRV. It may also be beneficial to explore the potential differences in these relationships based on sex, age, and fitness level.

HRV originates in the brain moving down to the cardiac vagal nerves that innervate the sinoatrial (SA) node. Thus, HRV cannot capture the complete autonomic function of the body and mainly represents cardiac pacemaker function (Hayano and Yuda 2019). Due to the cross-sectional nature of this study, directionality of the observed associations cannot be established. Individuals with lower HRV may be less likely to exercise and have poorer sleep due to autonomic dysfunction. The functioning of the autonomic nervous system can impact sleep homeostasis, while changes in sleep physiology may also disturb autonomic cardiovascular regulation (Tobaldini et al. 2013). Furthermore, there may be confounding factors that influence these relationships, such as genetics, training status, psychological state, environment, etc. (Tiwari et al. 2021). The small effect sizes and wide confidence intervals suggest that results should be interpreted with caution. However, even modest increases in HRV may be beneficial for health outcomes. The consistent statistical significance indicates that there is an effect that is non-random. Future research should replicate these findings and incorporate longitudinal analyses to better assess their biological relevance. The use of cross-sectional data can only capture associations and cannot conclude temporal or causal relationships. Future studies should validate these findings and explore how exercise type may affect the sleep–HRV relationship while incorporating more diverse sleep measures [e.g., polysomnography (PSG) and sleep diary], objective exercise measurements, and ecologically valid HRV measures.

## Conclusion

Findings from a nationally representative sample of adults reveal that higher levels of VPA are associated with increased HRV, whereas MPA shows no significant association. A novel contribution of this study is the observation that short sleep exacerbates this association. Specifically, obtaining < 6 h of sleep may amplify the negative associations of inadequate exercise on HRV. In contrast, sleeping 6 h or more appears to exert a moderating, and protective role for HRV, regardless of physical activity levels. Addressing both poor sleep and inadequate exercise may be a valuable approach to improve HRV, a key marker of cardiovascular health. The findings of this cross-sectional population study should be validated in future mechanistic research.

## Supplementary Information

Below is the link to the electronic supplementary material.

Supplementary file1 (DOCX 36 KB)

## Data Availability

This study was not formally registered on an official website, and purpose of data collection was described in the NIH under Grant No. U19-AG051426-07. The analysis plan was not formally pre-registered. De-identified data from this study are available in a public archive: https://www.icpsr.umich.edu/web/ICPSR/series/203. Analytic code used to conduct the analyses presented in this study are not available in a public archive. They may be available by emailing the corresponding author. All materials used to conduct the study are available in a public archive: https://www.icpsr.umich.edu/web/ICPSR/series/203 [1].
