# Supplementary material for: The interaction between exercise and sleep with heart rate variability: cross-sectional study
Source: Eur J Appl Physiol. 2025 Jul 5;126(1):223–37. doi: 10.1007/s00421-025-05887-y (PMC12881066; doi:10.1007/s00421-025-05887-y)
Supplement: Supplementary file 1 — Supplementary file1 (DOCX 36 KB) [file 421_2025_5887_MOESM1_ESM.docx]

**Title:** The Interaction Between Exercise and Sleep with Heart Rate Variability: Cross-Sectional Study

**Journal Name:** *European Journal of Applied Physiology*

**Authors:** Taylor Fein B.S.^1^, Muhammad Thalil Ph.D., Soomi Lee Ph.D.

**Institutional Affiliation:** ^1^Integrative and Biomedical Physiology, Huck Institutes of Life Sciences, The Pennsylvania State University, 101 Huck Life Sciences Building, University Park, PA 16802, USA

**Corresponding Author Contact Information:** Taylor Fein ([tpf5305@psu.edu](mailto:tpf5305@psu.edu))

**Supplementary Table 1**: Exercise Intensity Definitions

| VIGOROUS | -Which causes your heart to beat so rapidly you can feel it in your chest and you perform it long enough to work up a good sweat and breathe heavily (e.g., competitive sports, running, vigorous swimming, high intensity aerobics, digging in the garden, or lifting heavy objects.) |
| --- | --- |
| MODERATE | Which causes your heart rate to increase slightly and you typically work up a sweat  (e.g., leisurely sports like light tennis, slow or light swimming, low intensity aerobics or golfing without a power cart, brisk walking, mowing the lawn with a walking lawnmower). |
| LIGHT | Which requires little physical effort (e.g., light housekeeping like dusting or laundry,  bowling, archery, easy walking, golfing with a power cart or fishing) |
| Participants were then asked, “Keeping these definitions in mind, do you engage in regular exercise, or activity, of any type for 20 minutes or more at least 3 times/week?” If they answered yes, they were instructed to specific the exercise/activity, intensity, number of times per week, and average number of minutes per session. They were given the option of listing up to seven exercises/activities (Ryff et al. 2010). | |

**Supplementary Table 2:** Sample Descriptives and Tests of Group Differences Between Those Who Provided HRV Data and Those Who Did Not (*N*=1,255)

|  | **HRV Data Provided** | **HRV Data Unavailable** |  |
| --- | --- | --- | --- |
| **Characteristic** | **N=1156***^1^* | **N=99***^1^* | **p-value***^2^* |
| **Age (years)** | 57 (11) | 64 (12) | **<0.001** |
| *Min, Max* | 35, 86 | 36, 86 |  |
| **Sex** |  |  | 0.6 |
| *Male* | 497 (43%) | 45 (45%) |  |
| *Female* | 659 (57%) | 54 (55%) |  |
| **Marriage** |  |  | 0.4 |
| *Married* | 723 (63%) | 66 (67%) |  |
| *Unmarried* | 433 (37%) | 33 (33%) |  |
| **Race** |  |  | 0.7 |
| *Non-Hispanic White* | 882 (77%) | 77 (79%) |  |
| *Non-white* | 270 (23%) | 21 (21%) |  |
| **Level of Education** | 7.48 (2.51) | 7.34 (2.75) | 0.5 |
| **Chronic Conditions** | 3.26 (2.43) | 4.58 (2.75) | **<0.001** |
| **BMI (kg/m²)** | 30 (7) | 30 (7) | >0.9 |
| **CESD** | 9 (8) | 10 (9) | \|  \| 0.056 \| \| --- \| --- \| |
| **Smoker** |  |  | >0.9 |
| *No* | 607 (53%) | 51 (52%) |  |
| *Former* | 375 (32%) | 34 (34%) |  |
| *Yes* | 173 (15%) | 14 (14%) |  |
| **VPA** |  |  | **0.033** |
| *Adequate* | 244 (21%) | 12 (12%) |  |
| *Inadequate* | 912 (79%) | 87 (88%) |  |
| **MPA** |  |  | 0.9 |
| *Adequate* | 455 (39%) | 40 (40%) |  |
| *Inadequate* | 698 (61%) | 59 (60%) |  |
| **Sleep Quality** |  |  | >0.9 |
| *Good* | 560 (52%) | 48 (52%) |  |
| *Poor* | 520 (48%) | 44 (48%) |  |
| **Sleep Duration** |  |  | >0.9 |
| *Non-short* | 243 (59%) | 19 (59%) |  |
| *Short* | 166 (41%) | 13 (41%) |  |
| *Notes: CESD= Center for Epidemiological Studies Depression; BMI=body mass*  *index; kg=kilograms; m=meters, VPA=Vigorous Physical Activity, MPA=*  *Moderate Physical Activity*  *^1^*mean (SD) for continuous; n (%) for categorical | | | |
| *^2^*Wilcoxon rank sum test; Pearson's Chi-squared test | | | |

|  |
| --- |

**Supplementary Table 3:** Linear Model Results on the Association between Sleep Duration and HRV (N=391)

|  |  | | **HF-HRV ln (ms^2^)** | |  | | **RMSSD ln (ms)** | | | | | | |
| --- | --- | --- | --- | --- | --- | --- | --- | --- | --- | --- | --- | --- | --- |
|  | **Model 1** | | **Model 2** | | **Model 3** | | **Model 1** | | **Model 2** | | | **Model3** | |
|  | **B (95% CI)** | **p-value** | **B (95% CI)** | **p-value** | **B (95% CI)** | **p-value** | **B (95% CI)** | **p-value** | **B (95% CI)** | **p-value** | **B (95% CI)** | | **p-value** |
| Short Sleep Duration | 0.174  (-0.090, 0.440) | 0.197 | 0.064  (-0.201, 0.329) | 0.637 | 0.051  (-0.213, 0.316) | 0.703 | 0.111  (-0.013, 0.237) | 0.080 | 0.035  (-0.092, 0.162) | 0.589 | 0.020  (-0.106, 0.146) | | 0.754 |
| Age | — | — | -0.032  (-0.044, -0.021) | **<0.001** | -0.033  (-0.045, -0.020) | **<0.001** | — | — | -0.009  (-0.015, -0.004) | **0.001** | -0.010  (-0.016, -0.004) | | **0.002** |
| Women | — | — | 0.106  (-0.159, 0.371) | 0.435 | 0.138  (-0.131, 0.407) | 0.317 | — | — | 0.014  (-0.113, 0.141) | 0.826 | 0.029  (-0.100, 0.157) | | 0.662 |
| Unmarried | — | — | 0.079  (-0.203, 0.360) | 0.585 | 0.053  (-0.230, 0.336) | 0.712 | — | — | 0.075  (-0.059, 0.210) | 0.273 | 0.060  (-0.075, 0.194) | | 0.389 |
| Non-white | — | — | 0.410  (0.109, 0.711) | **0.008** | 0.452 (0.136, 0.767) | **0.005** | — | — | 0.223  (0.079, 0.367) | **0.003** | 0.212  (0.062, 0.363) | | **0.006** |
| Education | — | — | 0.023  (-0.027, 0.073) | 0.361 | 0.045  (-0.007, 0.096) | 0.093 | — | — | 0.009  (-0.015, 0.033) | 0.459 | 0.021  (-0.003, 0.014) | | 0.092 |
| Chronic Conditions | — | — | — | — | -0.020  (-0.071, 0.030) | 0.424 | — | — | — | — | -0.007  (-0.031, 0.018) | | 0.592 |
| BMI | — | — | — | — | 0.006  (-0.011, 0.024) | 0.500 | — | — | — | — | 0.005  (-0.003, 0.014) | | 0.219 |
| CESD | — | — | — | — | -0.019  (-0.036, -0.002) | **0.033** | — | — | — | — | -0.007  (-0.015, 0.001) | | 0.100 |
| Former Smoker | — | — | — | — | 0.387  (0.101, 0.673) | **0.008** | — | — | — | — | 0.210  (0.073, 0.346) | | **0.003** |
| Current smoker | — | — | — | — | 0.567  (0.181, 0.955) | **0.004** | — | — | — | — | 0.295  (0.109, 0.480) | | **0.002** |

Notes: CESD= Center for Epidemiological Studies Depression; BMI=body mass index; B=unstandardized beta coefficient; CI=confidence interval HF-HRV=high frequency HRV; RMSSD=root mean squared successive differences; ln=natural log, ms=milliseconds; All HRV measures were natural log-transformed

**Supplementary Table 4**: Linear model Results on the Association between Sleep Quality and HRV (N=1007)

|  |  | | **HF-HRV ln (ms^2^)** | |  | | **RMSSD ln (ms)** | | | | | | |
| --- | --- | --- | --- | --- | --- | --- | --- | --- | --- | --- | --- | --- | --- |
|  | **Model 1** | | **Model 2** | | **Model 3** | | **Model 1** | | **Model 2** | | | **Model 3** | |
|  | **B (95% CI)** | **p-value** | **B (95% CI)** | **p-value** | **B (95% CI)** | **p-value** | **B (95% CI)** | **p-value** | **B (95% CI)** | **p-value** | **B (95% CI)** | | **p-value** |
| Poor Sleep Quality | 0.038  (-0.120, 0.196) | 0.636 | -0.094  (-0.247, 0.059) | 0.229 | -0.007  (-0.174, 0.159) | 0.931 | 0.008  (-0.069, 0.084) | 0.846 | -0.051  (-0.128, 0.025) | 0.186 | -0.035  (-0.118, 0.048) | | 0.405 |
| Age | — | — | -0.033  (-0.040, -0.027) | **<0.001** | -0.031  (-0.039, -0.024) | **<0.001** | — | — | -0.010  (-0.013, -0.006) | **<0.001** | -0.008  (-0.011. -0.004) | | **<0.001** |
| Women | — | — | 0.104  (-0.049, 0.258) | 0.183 | 0.136  (-0.020, 0.291) | 0.088 | — | — | -0.007  (-0.084, 0.069) | 0.849 | 0.011  (-0.066, 0.089) | | 0.780 |
| Unmarried | — | — | 0.066  (-0.100, 0.232) | 0.436 | 0.050  (-0.117, 0.217) | 0.555 | — | — | 0.051  (-0.030, 0.133) | 0.220 | 0.035  (-0.048, 0.118) | | 0.409 |
| Non-white | — | — | 0.405  (0.212, 0.598) | **<0.001** | 0.417  (0.221, 0.613) | **<0.001** | — | — | 0.214  (0.117, 0.310) | **<0.001** | 0.213  (0.114, 0.312) | | **<0.001** |
| Education | — | — | -0.018  (-0.049, 0.012) | 0.239 | -0.013  (-0.044, 0.019) | 0.430 | — | — | -0.007  (-0.022, 0.008) | 0.367 | -0.003  (-0.018, 0.013) | | 0.717 |
| Chronic Conditions | — | — | — | — | -0.026  (-0.057, 0.006) | 0.108 | — | — | — | — | -0.013  (-0.028, 0.003) | | 0.110 |
| BMI | — | — | — | — | -0.007  (-0.019, 0.004) | 0.219 | — | — | — | — | -0.002  (-0.007, 0.004) | | 0.610 |
| CESD | — | — | — | — | -0.008  (-0.151, 0.192) | 0.138 | — | — | — | — | -0.0001  (-0.005, 0.005) | | 0.977 |
| Former Smoker | — | — | — | — | 0.021  (-0.151, 0.192) | 0.813 | — | — | — | — | -0.001  (-0.086, 0.085) | | 0.990 |
| Current smoker | — | — | — | — | 0.330  (0.095, 0.565) | **0.006** | — | — | — | — | -.158  (0.041, 0.275) | | **0.008** |

Notes: CESD= Center for Epidemiological Studies Depression; BMI=body mass index; B=unstandardized beta coefficient; CI=confidence interval HF-HRV=high frequency HRV; RMSSD=root mean squared successive differences; ln=natural log, ms=milliseconds; All HRV measures were natural log-transformed

References

Ryff CD, Seeman T, Weinstein M (2010) Midlife in the United States (MIDUS 2): Biomarker Project, 2004-2009: Archival Version
